# All-cause and cardiovascular mortality in relation to lung function in the full range of distribution across four Eastern European cohorts

**DOI:** 10.1038/s41598-022-17261-5

**Published:** 2022-07-28

**Authors:** Tatyana Court, Nadezda Capkova, Andrzej Pająk, Sofia Malyutina, Abdonas Tamosiunas, Martin Bobák, Hynek Pikhart

**Affiliations:** 1 Faculty of Science, Research Centre for Toxic Compounds in the Environment (RECETOX), Masaryk University, Kotlarska 2, Brno, Czech Republic; 2 Research Department of Epidemiology and Public Health, University College London, London, UK; 3 National Institute of Public Health, Prague, Czech Republic; 4 Department of Epidemiology and Population Sciences, Institute of Public Health, Jagiellonian University Medical College, Kraków, Poland; 5 Research Institute of Internal and Preventive Medicine – Institute of Cytology and Genetics, Siberian Branch of the Russian Academy of Sciences, Novosibirsk, Russia; 6 Novosibirsk State Medical University, Novosibirsk, Russia; 7 Laboratory of Population Research, Institute of Cardiology, Lithuanian University of Health Sciences, Kaunas, Lithuania

**Keywords:** Risk factors, Epidemiology, Respiratory tract diseases

## Abstract

It is unclear whether the dose–response relationship between lung function and all-cause and cardiovascular mortality in the Central and Eastern European populations differ from that reported in the Western European and American populations. We used the prospective population-based HAPIEE cohort that includes randomly selected people with a mean age of 59 ± 7.3 years from population registers in Czech, Polish, Russian and Lithuanian urban centres. The baseline survey in 2002–2005 included 36,106 persons of whom 24,944 met the inclusion criteria. Cox proportional hazards models were used to estimate the dose–response relationship between lung function defined as FEV1 divided by height cubed and all-cause and cardiovascular mortality over 11–16 years of follow-up. Mortality rate increased in a dose–response manner from highest to lower FEV1/height^3^ deciles. Adjusted hazard ratios (HR) of all-cause mortality for persons in the 8th best, the 5th and the worst deciles were 1.27 (95% CI 1.08‒1.49), 1.37 (1.18–1.60) and 2.15 (1.86‒2.48), respectively; for cardiovascular mortality, the respective HRs were 1.84 (1.29–2.63), 2.35 (1.67–3.28) and 3.46 (2.50‒4.78). Patterns were similar across countries, with some statistically insignificant variation. FEV1/height^3^ is a strong predictor of all-cause and cardiovascular mortality, across full distribution of values, including persons with preserved lung function.

## Introduction

The prognostic value of impaired lung function in association with mortality is well documented^1–6^. It has been shown that reduced levels of forced expiratory volume measured in one second (FEV1) are a better tool in predicting mortality than forced vital capacity (FVC)^7,8^. FEV1 was also an independent predictor of all-cause and cardiovascular mortality and morbidity in population-based studies^9,10^. Impaired FEV1 had a stronger relationship with mortality and cardiovascular events than other common risk factors even at modest range of decrease in FEV1^11^ independent from smoking and chronic respiratory diseases (COPD)^12,13^. Evidence is limited on predictive properties of FEV1 among persons with preserved lung function, but earlier studies suggested possible associations between lung function and mortality among those without lung function impairment^14–16^.

The conventional approach of lung function evaluation as the percentage of predicted value compared with reference values from healthy population has been criticised^17–19^. It less accounts for age-specific height variability and more prone to false positive results^19^. Global Lung Function Initiative (GLI) equation^20^ based on standardised Z-score by taking into accounts age, sex, height and ethnicity are a more valid approach but it has some limitations in elderly population^17,19^. Some other methods of standardising spirometry indices including FEV1 divided by height squared and/or cubed have been found a better alternative to predict survival function and mortality in this age category^17,21–24^. The assessment of lung function impairment by these methods is not dependent on predictive values and it is a more informative way of describing a dose–response relationship between lung function and mortality.

Most of the existing evidence in terms of mortality and lung function comes from Western European and American populations. Patterns of exposures and risk factors may be different in other populations, including Central and Eastern Europe. These countries have had higher mortality rates due to socioeconomic inequalities^25^, lifestyle patterns (e.g., nutritional, physiological and physical activity) and a very high burden from tobacco and alcohol use^26,27^. In this study we assessed the country-specific pattern of lung function defined as FEV1 divided by height cubed in association with all-cause and cardiovascular mortality in four Central and Eastern European countries.

## Results

### Baseline characteristics of the study population

**Figure 1:**
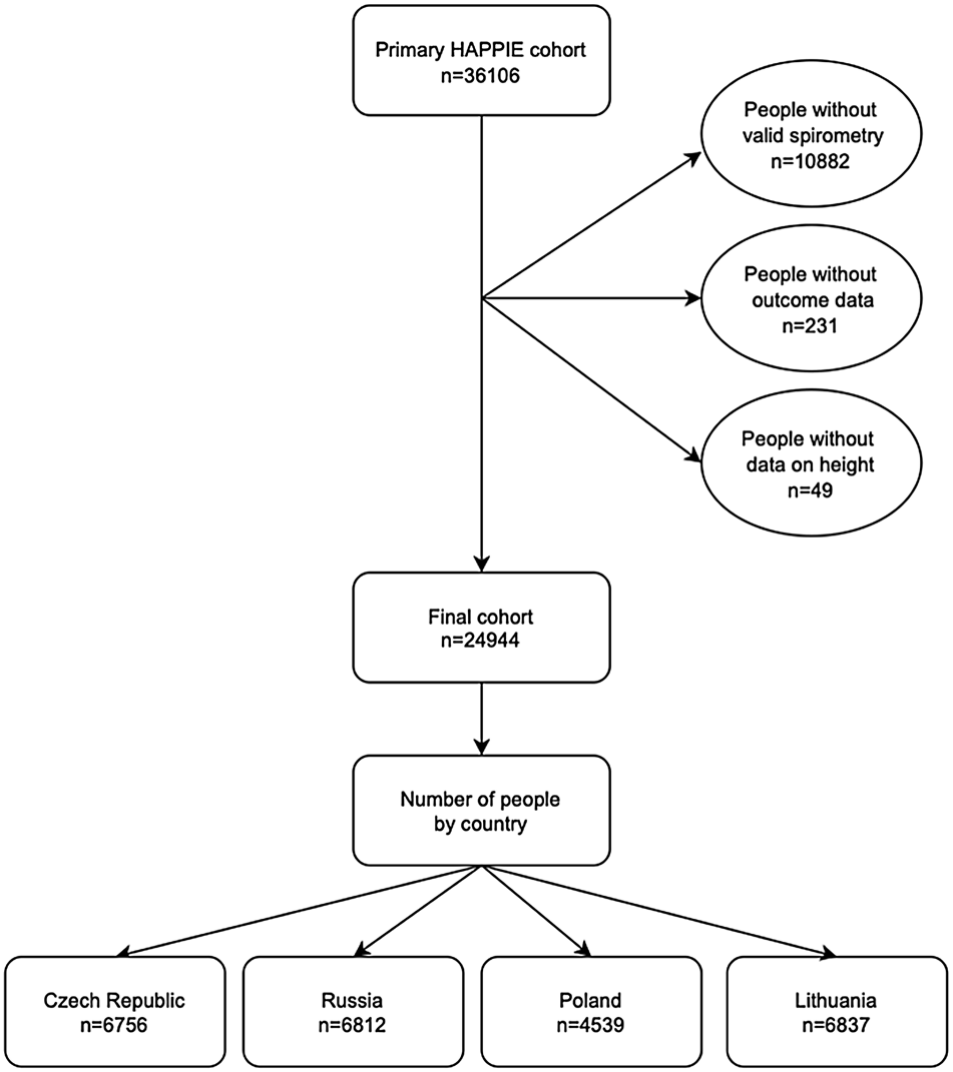
Flowchart of exclusion criteria of the HAPIEE study cohort.

Altogether 36,106 individuals were recruited at baseline, of whom 24,944 met the inclusion criteria (Fig. 1). For logistic reasons, spirometry was conducted on 25,224 persons. For the same reasons, in the Polish cohort spirometry tests were performed only on random 50% of respondents in the 2nd year of baseline survey. Another reason for missing spirometry data was due to non-response in clinical examinations in Czech and Polish participants. Compared with the top tertile of FEV1/height^3^, persons in the lowest tertile were those at older ages with highest proportion of women and people with smoking history. The number of underlying chronic cardiovascular and lung diseases and diabetes was also greater for this group (Table 1). In comparison between countries, the prevalence of people in the lowest FEV1/height^3^ tertile was highest in Czech Republic and Lithuania, while a third of the Russian population were in the highest tertile group (Table 1).

**Table 1:** Characteristics of the study sample by FEV1/height^3^ tertiles (n = 24,944).

	Lowest tertile	Intermediate tertile	Highest tertile
	(n = 8314)	(n = 8313)	(n = 8317)
Age (years), mean (SD)	61.8 (6.7)	58.9 (7.1)	55.8 (6.9)
**Age (years), %**			
< 50	6.6	13.8	25.7
50–59	28.9	39.8	46.6
60–69	56.6	42.0	25.5
≥ 70	7.9	4.3	2.1
Women, %	63.8	58.3	38.5
**Country, %**			
Czech Republic	32.0	27.2	22.0
Russia	22.3	26.7	33.0
Poland	18.9	18.4	17.3
Lithuania	26.8	27.7	27.7
**Occupational status, %**			
Employed	22.7	38.3	56.2
Retired/employed	12.9	13.8	12.8
Retired/unemployed	60.4	42.7	24.9
Unemployed	4.1	5.1	6.2
**Smoking status, %**			
Current, ≥ 1 cigarette	25.0	22.4	24.1
Current, < 1 cigarette	1.5	2.0	2.3
Past smoker	20.9	20.9	23.3
Never	52.6	54.6	50.2
**Smoking category**^**a**^**, %**			
Light	45.7	47.2	45.7
Moderate	44.0	43.5	44.5
Heavy	10.3	9.3	9.8
**Alcohol consumption**^**b**^**, %**			
Never	33.5	26.6	19.8
< 1/monthly	28.3	30.8	26.8
1–3/monthly	18.2	20.2	23.8
1–4/weekly	15.4	18.1	23.7
≥ 5/weekly	4.6	4.4	5.9
Deprivation range^c^, mean (SD)	2.3 (3.1)	2.1 (2.9)	2.0 (2.8)
Physical activity moderate^d^, mean (SD)	15.2 (11.8)	15.6 (11.5)	14.8 (11.1)
Physical activity vigorous^e^, mean (SD)	3.6 (5.8)	3.7 (5.6)	3.7 (5.6)
BMI, mean (SD), kg/m^2^	29.5 (5.6)	28.7 (4.9)	27.7 (4.4)
**Comorbidities, %**			
Cardiovascular diseases			
Hypertension	71.8	64.9	57.8
Myocardial infarction	9.4	6.7	4.8
Ischemic heart disease	16.9	12.4	8.9
Stroke	5.3	3.9	2.2
Lung diseases			
COPD	23.3	15.4	11.9
Asthma	8.1	3.2	1.9
Cough (> 3 months)	21.1	14.2	11.7
Chest pain (> 3 months)	18.7	13.4	11.8
Any type of cancer	7.0	5.7	3.5
Other diseases			
Diabetes	12.9	8.3	5.2
Any type of surgery	1.3	1.2	0.9

*BMI* body mass index, *COPD* chronic obstructive pulmonary disease.

^a^Smoking category ((current or past heavy smoker (> 30 cigarettes per day), moderate smoker (11–29 cigarettes per day), or light smoker (< 10 cigarettes per day)).

^b^Alcohol consumption (never, graduated frequency from 1–3 drinks monthly or 1–5 drinks weekly).

^c^Deprivation scale (graded from 1 as a least deprived up to 12 as a most deprived).

^d^Number of hours per week undertaken by household domain physical activity (e.g., housework, gardening, maintenance of the house etc.).

^e^Number of hours of vigorous physical activity per week (e.g., sports, play games and hiking).

### Association between lung function and all-cause and cardiovascular mortality

In total, 5069 persons died during a mean follow-up period of 13 years (Supplementary Table 1). More than a third of total deaths was due to cardiovascular causes. Mortality rate increased in a dose–response manner from the highest to lower FEV1/height^3^ deciles (mortality rate, 95% confidence interval (CI); 0.86, 0.76‒0.96 per 100 person-years among people in the highest and 3.10, 2.91‒3.31 per 100 person-years among people in the lowest FEV1/height^3^ decile (Table 2)). In the Cox proportional hazards regression model 1 that was adjusted for age, sex and country (Table 2, Supplementary Fig. 3), risk of death was already elevated in the group of people in the 8th decile (HR 1.39, 95% CI 1.19‒1.62) and it had a more than threefold increase in the lowest FEV1/height^3^ decile (HR 3.33, 95% CI 2.89‒3.82). In the model 2 that was adjusted for other baseline covariates (Table 2), being within the 8th decile of FEV1/height^3^ was still associated with increased mortality risk (HR 1.27, 95% CI 1.08‒1.49), and the risk was doubled in the lowest FEV1/height^3^ decile (HR 2.15, 95% CI 1.86‒2.48).

**Table 2:** Association between FEV1/height^3^ deciles and all-cause mortality.

Type of groups	No. of persons	No. of deaths	Person-years of follow-up	Deaths per 100 person-years (95% CI)	Model 1 adjusted HR^†^ (95% CI)	Model 2 adjusted HR^‡^ (95% CI)
Highest (10th decile)	2491	280	32,646	0.86 (0.76–0.96)	1.00	1.00
9th	2489	311	32,984	0.94 (0.84–1.05)	1.11 (0.94–1.31)	1.04 (0.88–1.23)
8th	2492	378	32,767	1.15 (1.04–1.28)	1.39 (1.19–1.62)	1.27 (1.08–1.49)
7th	2489	391	32,718	1.19 (1.08–1.32)	1.40 (1.20–1.63)	1.23 (1.05–1.45)
6th	2488	439	32,410	1.35 (1.23–1.49)	1.65 (1.42–1.92)	1.34 (1.14–1.56)
5th	2495	488	32,426	1.50 (1.38–1.64)	1.74 (1.50–2.02)	1.37 (1.18–1.60)
4th	2490	504	32,309	1.56 (1.43–1.70)	1.80 (1.55–2.09)	1.37 (1.18–1.60)
3d	2494	579	31,795	1.82 (1.68–1.98)	2.10 (1.81–2.43)	1.51 (1.30–1.76)
2nd	2491	720	31,299	2.30 (2.14–2.47)	2.47 (2.14–2.85)	1.65 (1.42–1.91)
Lowest (1st decile)	2491	934	30,097	3.10 (2.91–3.31)	3.33 (2.89–3.82)	2.15 (1.86–2.48)

*FEV1* forced expiratory volume in 1 s, *CI* confidence interval, *HR* hazard ratio.

^†^Adjusted for age, sex and country.

^‡^Adjusted for occupation and education; alcohol consumption, smoking status, level of physical activity and body mass index; history of hypertension, ischemic heart disease, myocardial infarction, stroke.

Cardiovascular mortality pattern was similar but with stronger association (Table 3, Supplementary Fig. 4). The strong association was observed even in the 9th decile compared to the 10th FEV1/height^3^ decile (Table 3, HR 1.62, 95% CI 1.12‒2.32) and it was approximately 3.5 times higher in the lowest FEV1/height^3^ decile (HR 3.46, 95% CI 2.50‒4.78) (Table 3).

**Table 3:** Association between FEV1/height^3^ deciles and CVD mortality.

Type of groups	No. of persons	No. of deaths	Person-years of follow-up	Deaths per 100 person-years (95% CI)	Model 1 adjusted HR^†^ (95% CI)	Model 2 adjusted HR^‡^ (95% CI)
Highest (10th decile)	2491	51	32,646	0.15 (0.11–0.20)	1.00	1.00
9th	2489	88	32,984	0.27 (0.22–0.33)	1.61 (1.14–2.28)	1.62 (1.12–2.32)
8th	2492	101	32,767	0.31 (0.25–0.37)	1.85 (1.32–2.60)	1.84 (1.29–2.63)
7th	2489	129	32,718	0.39 (0.33–0.47)	2.29 (1.66–3.18)	2.10 (1.49–2.97)
6th	2488	135	32,410	0.42 (0.35–0.49)	2.47 (1.79–3.42)	2.02 (1.43–2.85)
5th	2495	162	32,426	0.50 (0.43–0.58)	2.76 (2.01–3.80)	2.35 (1.67–3.28)
4th	2490	163	32,309	0.50 (0.43–0.59)	2.73 (1.99–3.76)	2.11 (1.50–2.96)
3d	2494	209	31,795	0.66 (0.57–0.75)	3.44 (2.51–4.70)	2.49 (1.79–3.47)
2nd	2491	258	31,299	0.82 (0.73–0.93)	4.02 (2.95–5.47)	2.69 (1.93–3.73)
Lowest (1st decile)	2491	342	30,097	1.14 (1.02–1.26)	5.60 (4.14–7.57)	3.46 (2.50–4.78)

*FEV1* forced expiratory volume in 1 s, *CI* confidence interval, *HR* hazard ratio.

^†^Adjusted for age, sex and country.

^‡^Adjusted for occupation and education; alcohol consumption, smoking status, level of physical activity and body mass index; history of hypertension, ischemic heart disease, myocardial infarction, stroke.

### Sensitivity analysis

Although the interactions between FEV1/height^3^ and country were not statistically significant, we also conducted analyses stratified by country. The excess risk of death in the lowest FEV1/height^3^ tertile was greater for people from Lithuania (HR 5.28, 95% CI 3.86‒7.24), whereas participants from the Czech Republic showed a two-times lower all-cause mortality (HR 2.46, 95% CI 1.82‒3.33) (Table 4, Supplementary Fig. 5). In terms of cardiovascular mortality, the highest increase in the risk of death was in Poland where people in the 9th decile of FEV1/height^3^ range had more than a threefold increased risk of mortality (Table 5, Supplementary Fig. 6, HR 3.38, 95% CI 1.25‒9.16) and it was more than 10-times higher in the lowest FEV1/height^3^ decile (HR 11.1, 95% CI 4.36‒28.23) (Table 5, Supplementary Fig. 6). The country-specific confidence intervals were wide and the differences in HRs between countries were not statistically significant (p for country heterogeneity = 0.31).

**Table 4:** Association between FEV1/height^3^ deciles and all-cause mortality by country.

Type of groups	Adjusted (age + sex) HR (95% CI)	Adjusted (age + sex) HR (95% CI)	Adjusted (age + sex) HR (95% CI)	Adjusted (age + sex) HR (95% CI)
Country	Czech Republic (n = 6756)	Russia (n = 6812)	Poland (n = 4539)	Lithuania (n = 6837)
Highest (10th decile)	1.00	1.00	1.00	1.00
9th	0.85 (0.58–1.23)	1.22 (0.92–1.62)	1.31 (0.88–1.95)	1.41 (0.98–2.02)
8th	1.16 (0.82–1.62)	1.26 (0.95–1.66)	1.29 (0.86–1.92)	1.99 (1.41–2.80)
7th	1.41 (1.02–1.95)	1.77 (1.35–2.31)	1.42 (0.96–2.11)	1.74 (1.22–2.46)
6th	1.45 (1.05–2.02)	1.40 (1.07–1.85)	1.78 (1.21–2.60)	2.14 (1.52–3.02)
5th	1.65 (1.20–2.27)	1.83 (1.41–2.39)	1.72 (1.17–2.52)	2.58 (1.85–3.60)
4th	1.50 (1.09–2.06)	1.61 (1.23–2.10)	1.86 (1.28–2.71)	2.56 (1.83–3.59)
3d	1.77 (1.29–2.42)	1.97 (1.52–2.55)	2.08 (1.43–3.04)	2.94 (2.11–4.10)
2nd	2.01 (1.48–2.74)	2.51 (1.95–3.23)	2.29 (1.58–3.31)	3.48 (2.51–4.83)
Lowest (1st decile)	2.46 (1.82–3.33)	3.44 (2.69–4.39)	3.79 (2.66–5.40)	5.28 (3.86–7.24)

*FEV1* forced expiratory volume in 1 s, *CI* confidence interval, *HR* hazard ratio.

**Table 5:** Association between FEV1/height^3^ deciles and CVD mortality by country.

Type of groups	Adjusted (age + sex) HR (95% CI)	Adjusted (age + sex) HR (95% CI)	Adjusted (age + sex) HR (95% CI)	Adjusted (age + sex) HR (95% CI)
Country	Czech Republic (n = 6756)	Russia (n = 6812)	Poland (n = 4539)	Lithuania (n = 6837)
Highest (10th decile)	1.00	1.00	1.00	1.00
9th	2.41 (1.09–5.33)	1.38 (0.67–2.85)	3.38 (1.25–9.16)	1.70 (0.92–3.13)
8th	2.85 (1.32–6.14)	1.04 (0.48–2.25)	3.14 (1.15–8.57)	1.74 (0.94–3.22)
7th	3.79 (1.80–8.01)	2.11 (1.08–4.15)	3.43 (1.26–9.32)	2.43 (1.36–4.35)
6th	3.19 (1.49–6.82)	1.50 (0.74–3.06)	4.49 (1.68–11.96)	2.69 (1.51–4.81)
5th	2.98 (1.39–6.37)	2.58 (1.34–4.95)	5.38 (2.06–14.06)	3.70 (2.12–6.43)
4th	3.36 (1.59–7.08)	1.69 (0.84–3.39)	4.02 (1.50–10.76)	3.80 (2.17–6.66)
3d	4.61 (2.21–9.61)	2.44 (1.28–4.68)	5.24 (1.98–13.85)	3.99 (2.29–6.96)
2nd	4.33 (2.08–9.02)	3.06 (1.62–5.78)	6.84 (2.64–17.72)	5.09 (2.94–8.79)
Lowest (1st decile)	5.88 (2.85–12.14)	4.79 (2.60–8.84)	11.1 (4.36–28.23)	7.19 (4.21–12.28)

*FEV1* forced expiratory volume in 1 s, *CI* confidence interval, *HR* hazard ratio.

## Discussion

Using data from the population-based cohort of people from four Central and Eastern European countries, we found a robust dose–response relationship between lung function (defined as FEV1/height^3^) and all-cause mortality with even stronger effects on cardiovascular mortality. The association was seen throughout all levels of lung function even in people with preserved function. The observed differences between countries were non-significant with higher all-cause mortality noticed in Lithuania and strongest association with cardiovascular mortality in Poland.

Many previous studies investigated the link between impaired lung function and mortality^1–6^. The predicting properties of lung function in terms of mortality have been evaluated applying different approaches, and the choice of the best one is not straightforward. Lung function prediction equations based on reference values from the general population (FEV1% percent predicted and z-score) have been largely used for categorisation of airway obstruction, however, both have some limitations for the use in the elderly population^17–20^. The major pitfalls of these approaches arise from the lack of sufficient data on this age group, possible survival bias of the selected people and impact of comorbidities on body size^19^. In our study, we explored the trend in mortality risk and lung function defined as FEV1 divided by height cubed. It has been shown that FEV1 standardised with height is a valid approach for predicting mortality risk in the elderly population^17,23,24,28^. Studies based on FEV1 standardised with height are scarce and the majority of them assessed the mortality risk through the commonly used comparison between healthy and people with the impaired lung function from the lowest tertile or quartile^17,21,22,29^.

We ranked FEV1/height^3^ into deciles in order to explore the risk of death across all levels of lung function including individuals with preserved lung function. The association was continuous, graded and considerably stronger for deaths from cardiovascular causes than for all-cause mortality. There is a paucity of studies evaluated the lung function across the whole spectrum in association with mortality. Ashley et al., using data on 2869 subjects from the Framingham Study, found a continuous association between FVC and all-cause mortality^14^. Analyses of data on 1195 people of the Buffalo Health Study and 1541 participants of the Rancho Bernardo Study also reported associations with mortality across FEV1 quantiles and quartiles, respectively^15,16^. More recently, Gupta et al. in the study of lifelong non-smokers has shown a consistent association with the risk of death per unit decrement in FEV1 and FVC z-scores as a measure of lung function but in more detailed assessment, the association across lung function quartiles was observed only with deaths from cardiovascular causes^12^. Moreover, the risk increase was more pronounced in groups corresponding to mild and mild to moderate decline in lung function compared to more severe impairment. Similar trend was also found in another study on mortality and cardiovascular adverse events in association with reduced lung function; which suggested that existing thresholds in defining lung function impairment might be misleading in terms of real impact of lung function on health risks^11^.

In line with previous studies, we also found a stronger association with cardiovascular mortality compared to deaths from all-causes. The exact mechanism underlying these associations remains unclear. Some epigenetic aging biomarkers may reflect lung function in elderly people^30^. Previous studies demonstrated that impaired lung function was associated with the severity of coronary atherosclerosis and vascular stiffness^31,32^. It was also an independent risk factor for predicting future cardiovascular disease and diabetes^16,33,34^. Lung function might act via similar mechanisms as other common risk factors (i.e., hypertension, obesity, level of physical activity and smoking) by triggering inflammatory processes that play a causal role in the development of chronic diseases leading to increased mortality^22,35,36^. In our study, the graded association with the risk of death remained strong after adjusting for potential confounders across all deciles suggesting the direct causal effect of lung function with mortality.

Most of the previous studies were performed in western populations, while studies investigating other populations are limited. In the large prospective international multi-ethnic (PURE) cohort study, the risk of death and cardiovascular events was elevated in persons with impaired lung function across all populations and it was highest in people from low- and middle-income countries^11^.

In our study, the association was similar across four countries with different socioeconomic status and health behaviour patterns, with non-significant variation between the countries. Compared to other populations from the Czech Republic and Russia, participants from Poland and Lithuania had higher number of pre-existing conditions that might partly explain the variation in results (Supplementary Table 2). Interestingly, the number of heavy smokers and people with COPD was the highest in Russia, where the effect of lung function was not particularly strong. Previous analyses have shown that lower socioeconomic status throughout a lifespan was associated with poorer lung function^37^ and that larger socioeconomic inequalities and higher mortality risk were in Russia^25^. Our study confirmed that lung function is a strong independent predictor of mortality risk, while its contribution to variation in mortality between countries remains unclear.

## Strengths and limitations

Our study included people from urban communities of four Central and Eastern European countries. While the study cohort is in general representative for urban populations, it does not include rural areas, and the results are thus not generalizable to entire population of included countries. The response rate was comparable and follow-up time was balanced between countries. Although, some participants with less healthy status from Czech and Polish cohorts did not underwent the baseline clinical examination and that could lead to the underestimation of our results. Large sample size, standardised data collection across all populations, adjustment for a large number of investigating covariates in the association of lung function and mortality is the particular strength of this study. However, the self-report of information in the questionnaire may be a source of reporting bias.

Limited data is available on the use of FEV1/height^3^ in defining lung function and further studies needed to validate this approach. However, such an approach does not require reference values and considered as a better tool for predicting mortality risk in the elderly population^17,23,24,28^.

Finally, the nature of our design cannot entirely exclude reverse causation, although the longitudinal design, extensive covariate adjustment of models and long follow-up likely to minimize these limitations.

## Conclusion

This study explored the association of lung function with all-cause and cardiovascular mortality in the Central and Eastern European populations. We showed a significant dose–response relationship of lung function with risk of death across the whole spectrum of FEV1/height^3^. The results emphasize the important contribution of lung function to cardiovascular and total mortality risk. If further research confirms the use of FEV1/height^3^ in assessing lung function in the elderly population, this measure may have a useful role in predicting future mortality risk in clinical practice.

## Methods

### Study design and participants

The prospective Health, Alcohol and Psychosocial factors in Eastern Europe (HAPIEE) cohort study has been designed to investigate risk factors for high rates of mortality and cardiovascular diseases in four Central and Eastern European countries (e.g., Czech Republic, Poland, Russia and Lithuania)^38^. It includes randomly selected people with a mean age of 59 ± 7.3 from population registers in seven towns in the Czech Republic and in big cities such as Novosibirsk in Russia, Krakow in Poland and Kaunas in Lithuania (N = 36,106). Data on age, sex, health status, medical examination, lifestyle, socioeconomic and psychosocial factors were collected during 2002–2005. The follow-up survey in the Czech Republic, Russia and Poland and baseline survey in Lithuania were conducted in 2005–2008 with the use of face-to-face computer assisted personal interviews combined with the clinical examination.

The follow-up time was estimated based on deaths occurring until the end of 2020 in the Czech Republic, until 31 July 2017 in Poland, until the end of 2017 in Russia and until 31 March 2019 in Lithuania. Persons with complete follow-up data were included in the study. Participants were censored on the date of death or the end of the study depending on data availability for each country.

### Ethics approval

All participants provided written informed consent. The study was performed in line with the principles of the Declaration of Helsinki. Approval was granted by the Joint UCL/UCLH Committees on the Ethics of Human Research (Committee Alpha), reference 99/0081; the Ethical Committee of the Institute of Internal Medicine, Siberian Branch of the Russian Academy of Medical Sciences, March 14, 2002 (Protocol No. 1); the Ethics Committee of the Kaunas Medical University (reference P1-09/2005); and Ethics Committee at the National Institute of Public Health, Prague (reference 2002-01-08/P1).

### Spirometry

Lung function test was performed using a Micro-Medical Microplus spirometer. Participants with acute pulmonary infections and illnesses (e.g., vomiting and nausea), recent surgical procedures and cardiovascular conditions (e.g., myocardial infarction and stroke) were excluded from testing^39^. Two or more measurements of FEV1 within 150 ml variation considered for the study^39^. For each participant the highest value of FEV1 was selected. FEV1 was standardised by height and defined as FEV1 divided by height cubed (FEV1/height^3^). It was expressed at levels of tertiles and deciles of their distribution for further analyses.

### Outcome

The primary outcome was all-cause mortality. Dates of death were obtained from the national or regional (Novosibirsk) death registers in each country. All registers have been shown a complete coverage of deaths^38^. In this study cause-specific mortality was based on underlying causes of death which are determined according to the selection and application rules of ICD-10 maintained by the World Health Organization (WHO).

### Covariates

Data on covariates was obtained from questionnaires and medical examination. The selection of variables was based on their known association with mortality^40,41^. For the adjustment we considered age, sex, country, education (primary, secondary education, college or university degree), occupation (employed as entrepreneur or freelancer, housewife, farmer, retired or unemployed), deprivation scale (graded from 1 as a least deprived up to 12 as a most deprived), smoking status (never, current or past heavy smoker (> 30 cigarettes per day), moderate smoker (11–29 cigarettes per day), or light smoker (< 10 cigarettes per day))^42^, alcohol consumption (never, graduated frequency from 1 to 3 drinks monthly or 1–5 drinks weekly) and physical activity (as number of hours demanding physical activity per week). We also identified the following self-reported comorbidities: stroke, myocardial infarction, ischemic heart disease, hypertension (defined as measured blood pressure > 140/90 mm Hg and self-reported treated hypertension), diabetes (treated and/or untreated), asthma and COPD, any type of surgery (in last 3 months), cancer.

Information on pulmonary symptoms such as cough with or without phlegm (for 3 months) and chest pain were also included in the analyses as markers of respiratory diseases. Confounders obtained during medical examination were weight, height, body-mass index (BMI), blood pressure and cholesterol level.

### Statistical analyses

All analyses were performed with Stata (Version 14; StataCorp). Descriptive statistics are presented as means with standard deviations (SD) or frequencies with proportions and were compared across FEV1/height^3^ tertiles.

The risk of death in association with FEV1/height^3^ was compared by Cox proportional hazards regression models. Deciles of FEV1/height^3^ were entered into the model as categorical variable accounting for level of lung function with highest category set as a reference. We used robust variance estimator to account for possible interactions between groups and multiple comparison. Proportional hazards assumptions were confirmed by exploring parallelism of log negative and log estimated survival curves for each covariate (Supplementary Figs. 1, 2). Hazard ratios (HR) with their corresponding 95% confidence intervals (CI) were estimated by crude (included age, sex and country) and confounder-adjusted models.

Dose–response relationship between FEV1/height^3^ and mortality was also assessed by entering FEV1/height^3^ as a continuous variable into the model. Linear trend was explored by adding quadratic terms and splines.

In addition to all-cause mortality, we investigated cardiovascular mortality. These analyses were restricted to diseases of circulatory system (ICD10 codes I00-I99).

We also conducted separate stratified sensitivity analysis for each country.

## Supplementary Information

Supplementary Information.

## Data Availability

The data that support the findings of this study are available from the register maintainers but restrictions apply to the availability of these data, which were used under license for the current study, and so are not publicly available. Data are however available from the authors upon reasonable request and with permission of the register maintainers.
