# Supplementary material for: All-cause and cardiovascular mortality in relation to lung function in the full range of distribution across four Eastern European cohorts
Source: Sci Rep. 2022 Jul 28;12:12959. doi: 10.1038/s41598-022-17261-5 (PMC9334616; doi:10.1038/s41598-022-17261-5)

Supplementary Table 1. Survival data by country.

Country	No. of persons	No. of total deaths	No. of CVD deaths	Person-years of follow-up	Mean Follow-up time (years)	Median Follow-up time (years)
Czech Republic	6 756	1 484	553	106 043	15.7	16.9
Russia	6 812	1 581	292	83 492	12.3	13.5
Poland	4 539	794	244	57 302	12.5	13.5
Lithuania	6 837	1 210	553	75 252	10.9	11.6

Supplementary Table 2. Characteristics of the study sample by country (n=24 944)

	Czech Republic (n=6 756)	Russia (n=6 812)	Poland (n=4 539)	Lithuania (n=6 837)
Age (years), mean (SD)	58.3 (7.1)	57.9 (7.0)	58.1 (6.9)	60.9 (7.6)
Age (years), %				
< 50	16.6	17.3	16.2	11.9
50-59	37.7	43.2	42.5	31.8
60-69	44.0	38.0	39.4	43.3
≥ 70	1.7	1.5	1.9	13.0
Women, %	54.2	54.0	50.6	54.5
Occupational status, %				
Employed	43.4	36.6	35.8	39.7
Retired/employed	8.0	18.5	5.9	17.5
Retired/unemployed	45.2	39.4	51.9	37.2
Unemployed	3.4	5.5	6.4	5.6
Smoking status, %				
Current, ≥ 1 cigarette	23.0	28.2	28.3	17.4
Current, < 1 cigarette	2.7	1.0	2.0	2.1
Past smoker	29.7	13.4	28.2	17.9
Never	44.6	57.3	41.5	62.6
Smoking category[a], %				
Light	55.4	36.3	43.7	45.6
Moderate	38.2	51.2	44.1	45.2
Heavy	6.4	12.5	12.2	9.2
Alcohol consumption[b], %				
Never	11.7	14.5	35.1	47.4
<1/monthly	25.9	37.6	21.8	26.7
1-3/monthly	21.1	21.6	0.2	19.9
1-4/weekly	28.5	23.5	19.1	5.5
≥5/weekly	12.9	2.7	3.8	0.5
Deprivation range[c], mean (SD)	1.6 (2.3)	3.8 (3.6)	2.1 (2.9)	1.0 (1.9)
Physical activity moderate[d], mean (SD)	13.7 (12.2)	16.8 (11.7)	13.9 (10.6)	15.8 (10.8)
Physical activity vigorous[e], mean (SD)	4.4 (5.4)	2.5 (5.9)	5.6 (6.1)	2.9 (4.8)
BMI, mean (SD), kg/m^2	28.2 (4.6)	28.5 (5.5)	28.3 (4.6)	29.4 (5.3)
**Comorbidities, %**				
Cardiovascular diseases				
Hypertension	65.2	65.6	60.5	66.5
Myocardial infarction	5.0	7.2	8.3	7.8
Ischemic heart disease	8.2	15.7	19.0	9.8
Stroke	3.3	4.8	2.5	4.2
Lung diseases				
COPD	14.4	23.3	11.3	16.3
Asthma	4.6	3.0	6.8	3.9
Cough (>3 months)	14.0	17.5	16.6	14.8
Chest pain (>3 months)	12.5	15.5	15.3	15.5
Any type of cancer	6.3	2.9	5.1	7.2
Other diseases				
Diabetes	11.4	5.2	12.1	7.6
Any type of surgery	2.0	0.2	1.9	1.0
Spirometry, mean (SD)				
FEV1	2.55 (0.8)	2.58 (0.8)	2.55 (0.8)	2.63 (0.8)
FVC	3.3 (0.9)	3.1 (0.9)	3.1 (0.9)	3.3 (0.9)
FEV1/height^3, l/m^2	0.53 (0.13)	0.58 (0.13)	0.55 (0.12)	0.56 (0.12)

BMI, body mass index; COPD, chronic obstructive pulmonary disease; FEV1, forced expiratory volume in 1 second; FVC, forced vital capacity.

[a]Smoking category ((current or past heavy (>30 cigarettes per day), moderate (11 - 29 cigarettes per day), or light (<10 cigarettes per day)).

[b]Alcohol consumption (never, graduated frequency from 1-3 drinks monthly or 1-5 drinks weekly).

[c]Deprivation scale (graded from 1 as a least deprived up to 12 as a most deprived).

[d]Number of hours per week undertaken by household domain physical activity (e.g., housework, gardening, maintenance of the house etc).

[e]Number of hours of vigorous physical activity per week (e.g., sports, play games and hiking)

Supplementary Figure 1. K-M curves of all-cause mortality and FEV1/height^3 deciles.

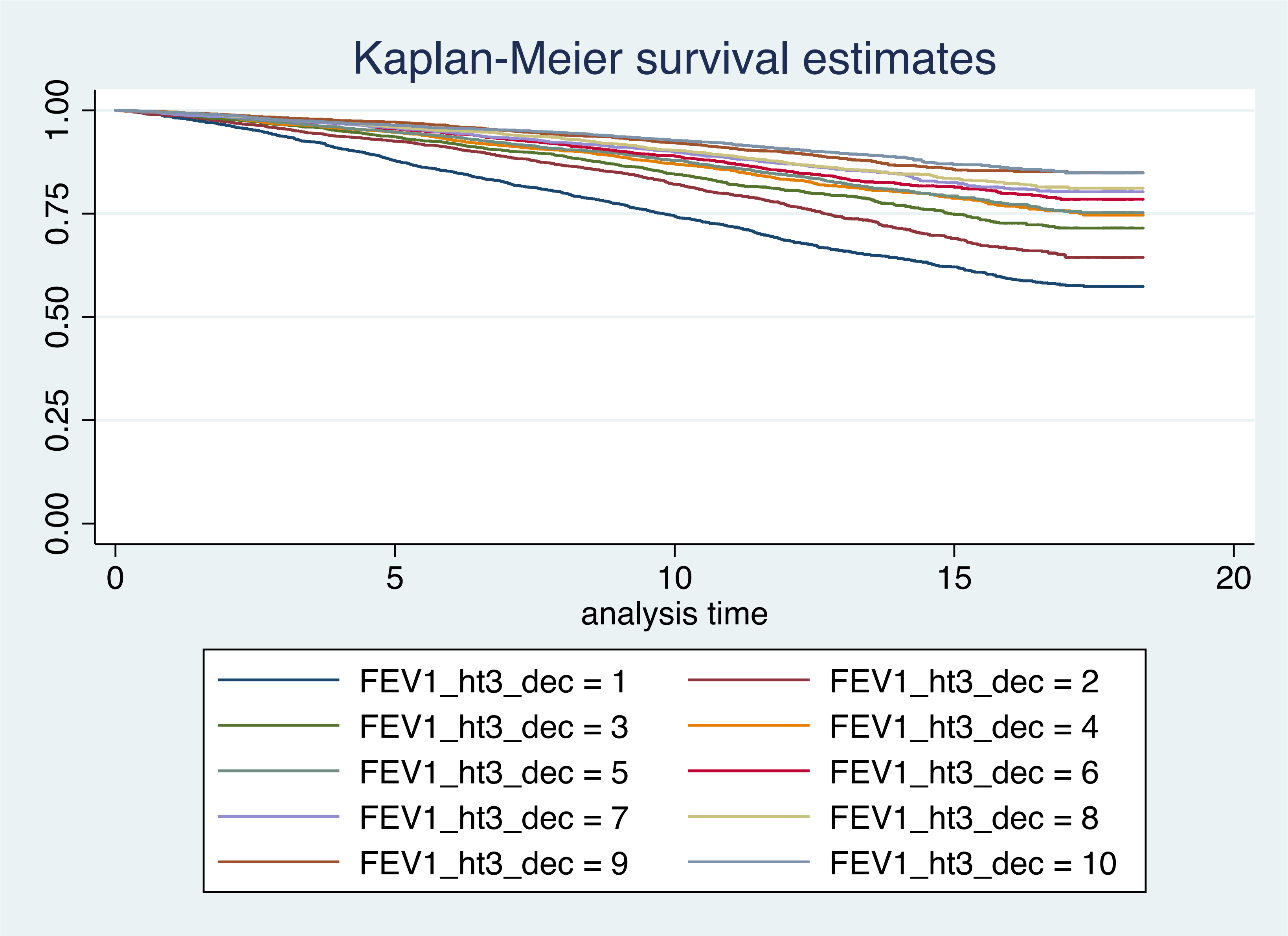

Supplementary Figure 2. K-M curves of CVD mortality and FEV1/height³ deciles.

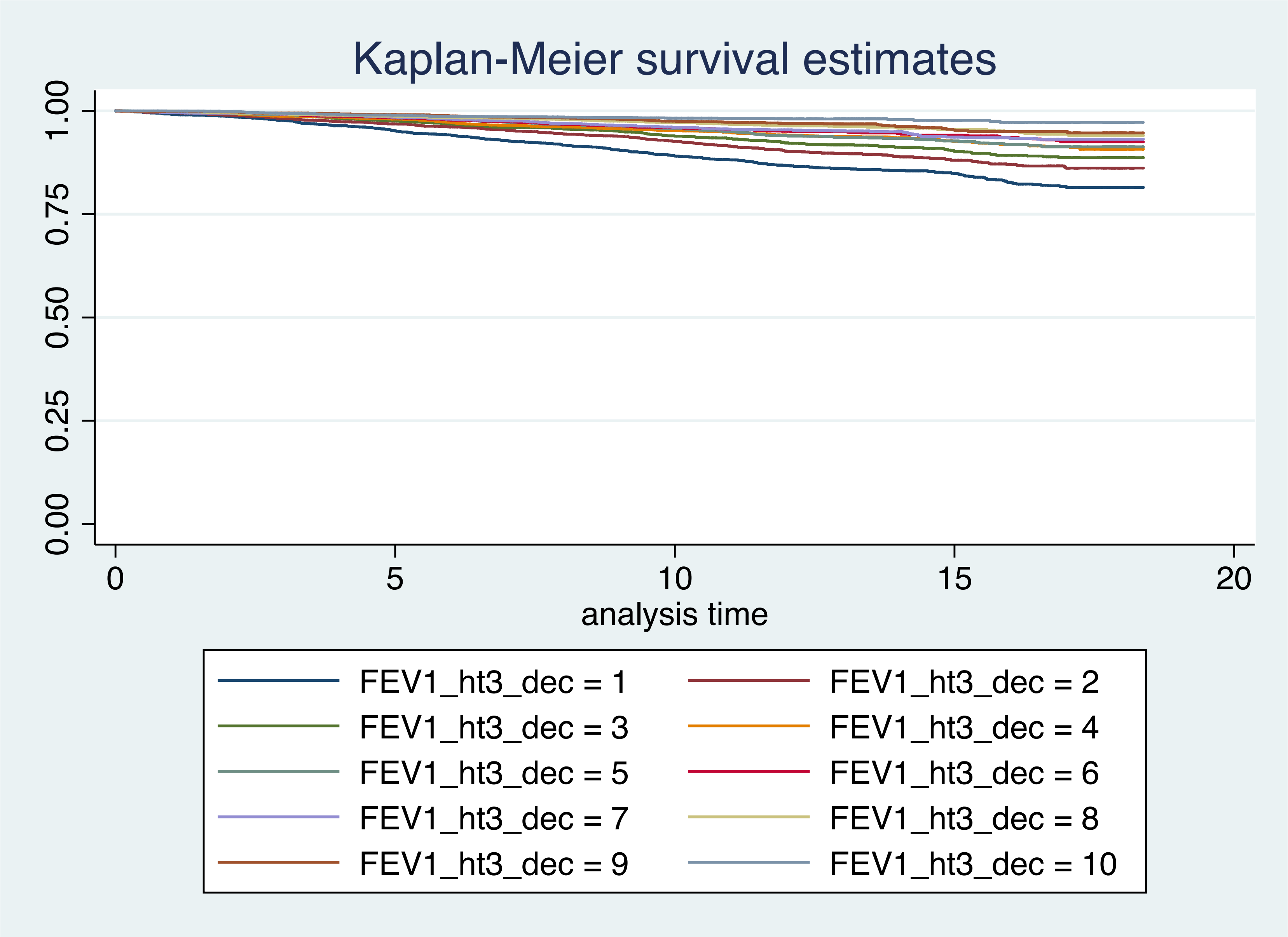

Supplementary Figure 3. FEV1/height^3 trend in all-cause mortality

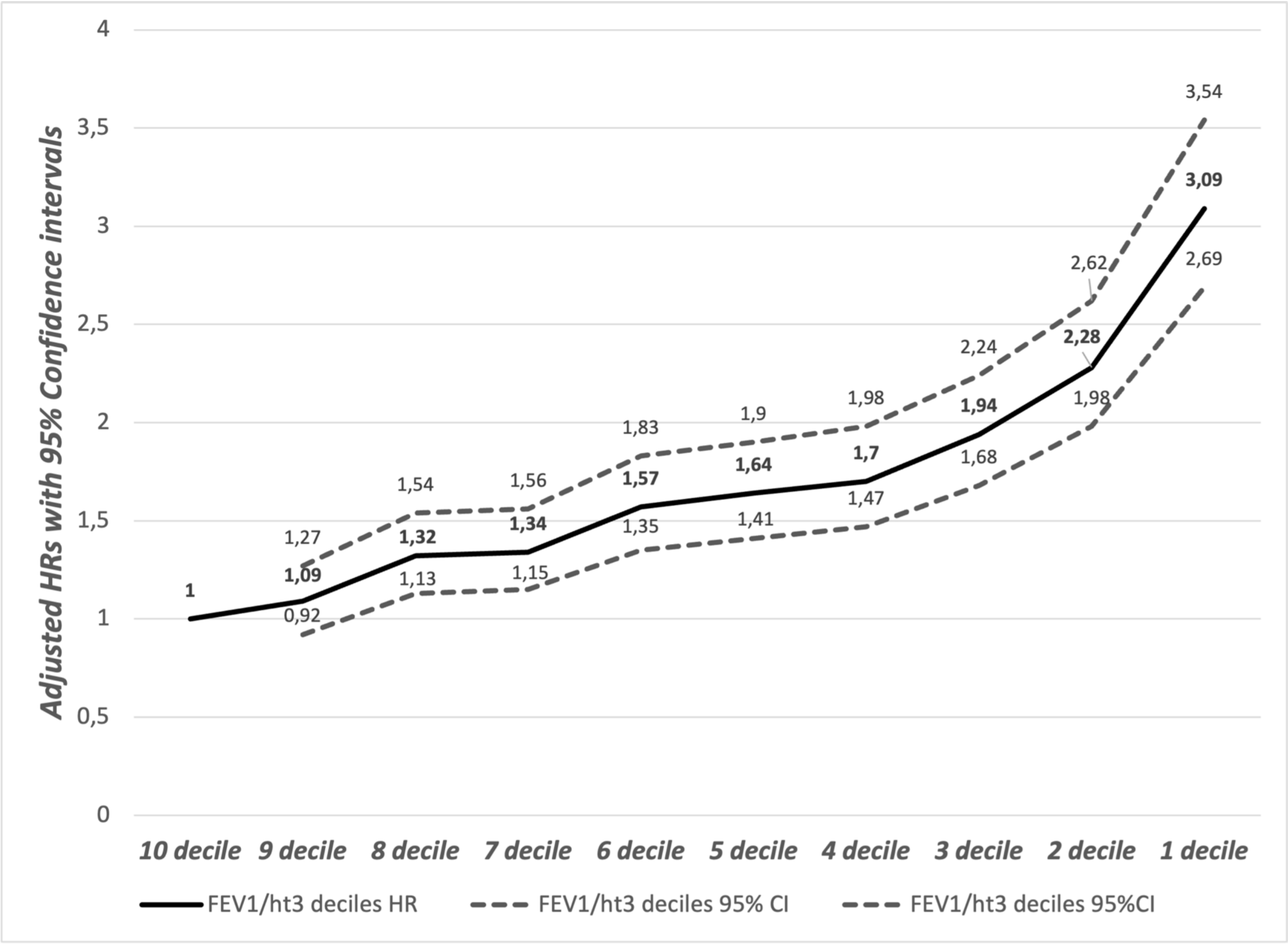

Supplementary Figure 4. FEV1/height^3 trend in CVD mortality

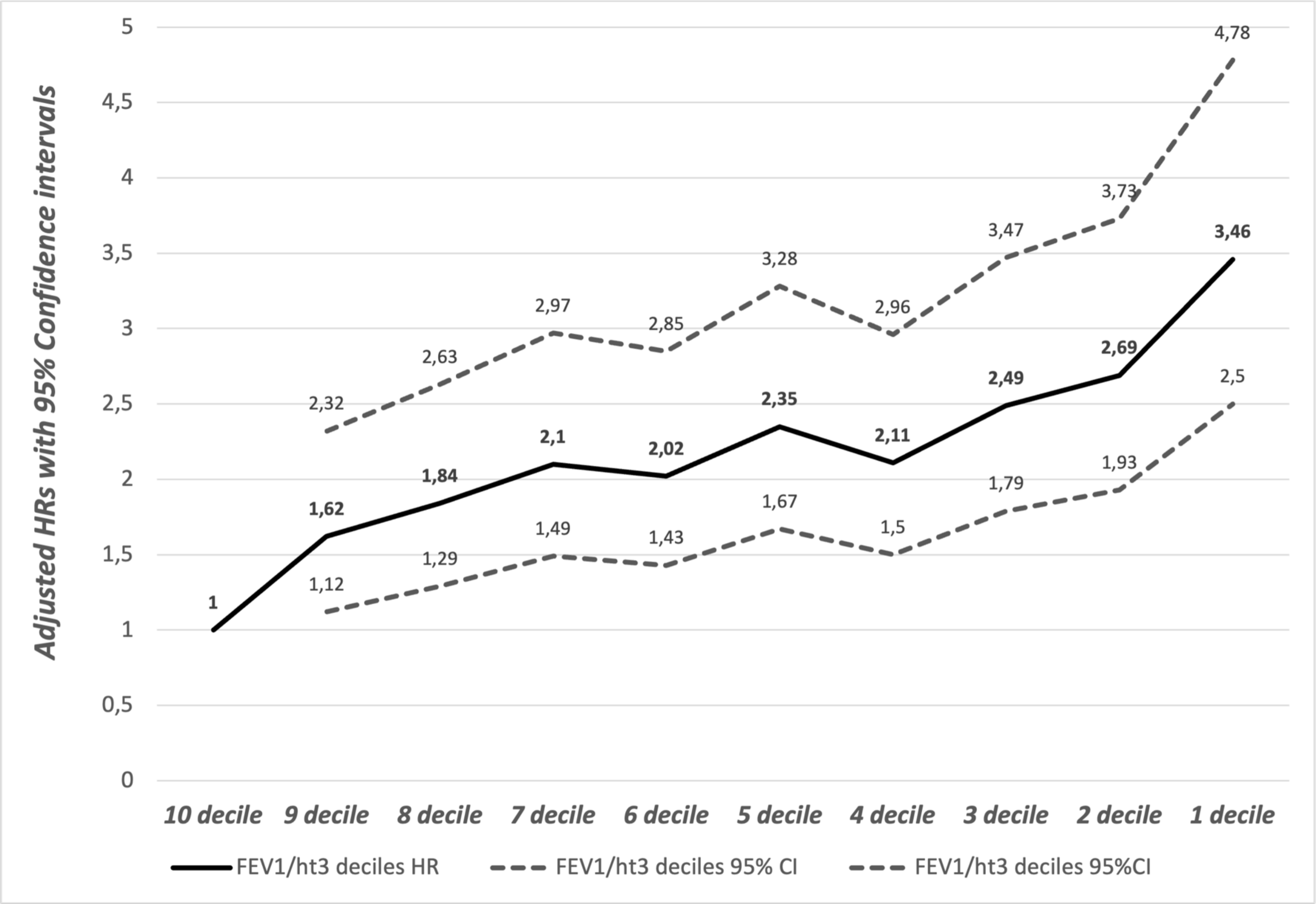

Supplementary Figure 5. Relative risk of all-cause mortality across FEV1/height³ deciles (reference highest 10th decile) by country (adjusted for occupation and education; alcohol consumption, smoking status, level of physical activity and body mass index; history of hypertension, ischemic heart disease, myocardial infarction, stroke).

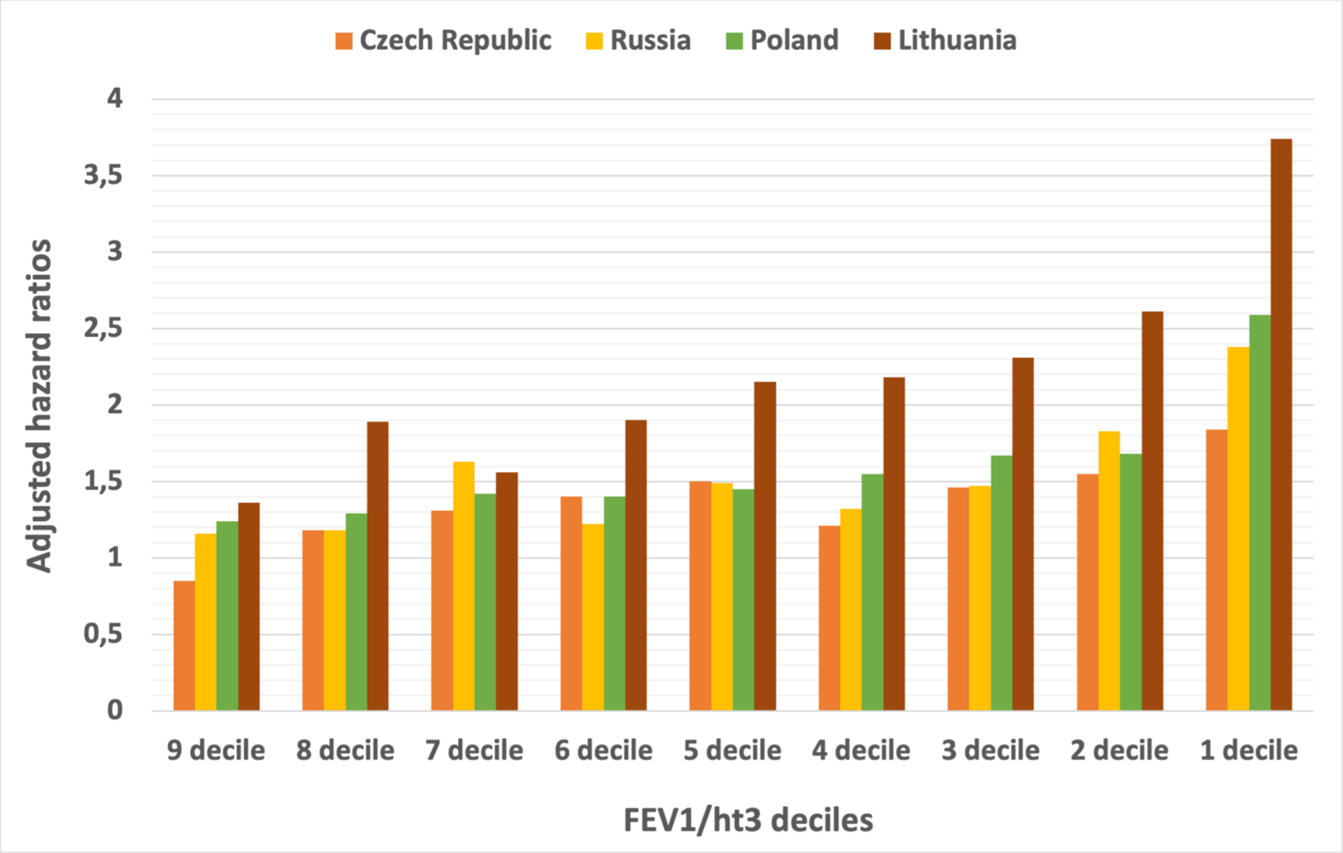

Supplementary Figure 6. Relative risk of CVD mortality across FEV1/height³ deciles (reference highest 10th decile) by country adjusted for occupation and education; alcohol consumption, smoking status, level of physical activity and body mass index; history of hypertension, ischemic heart disease, myocardial infarction, stroke).

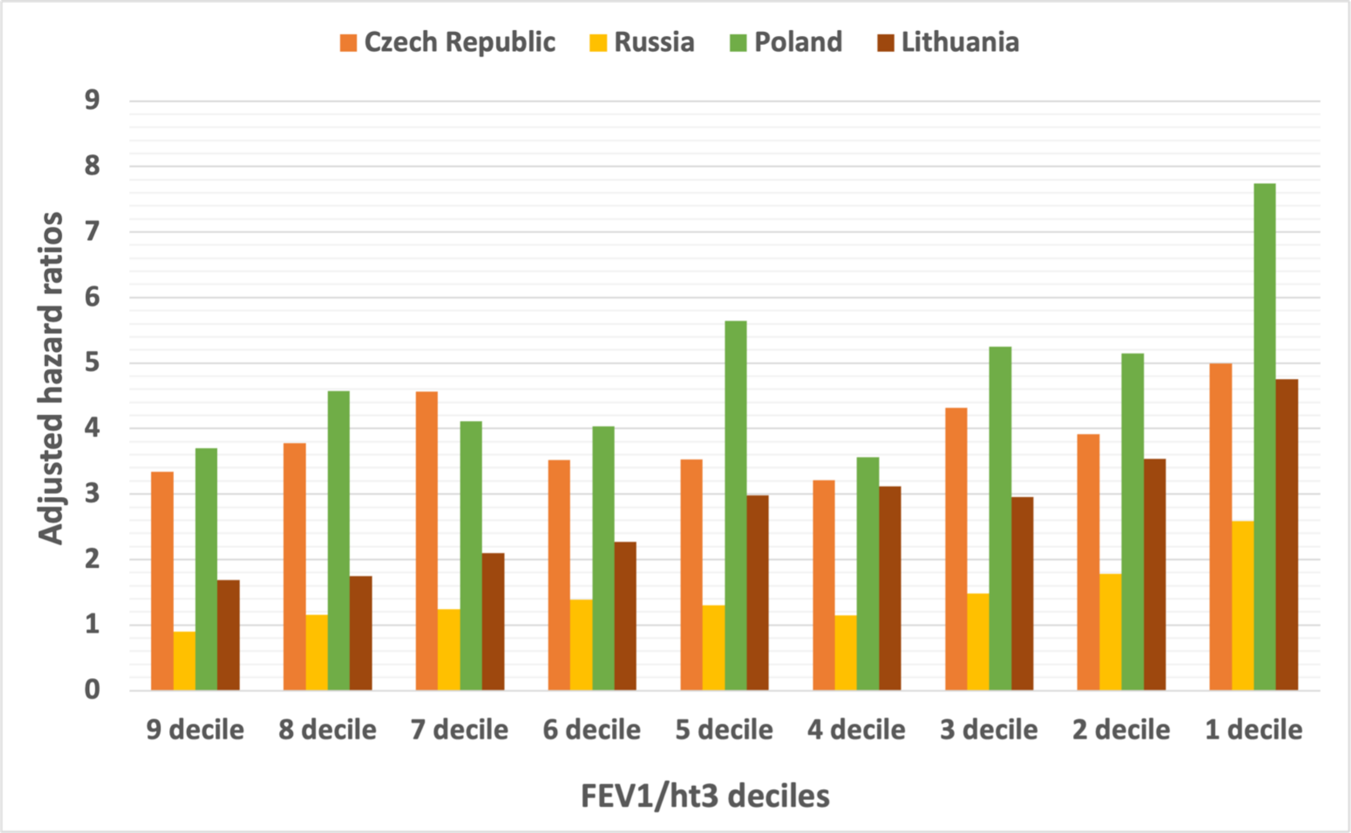

Supplement: Supplementary file 1 — Supplementary Information. [file 41598_2022_17261_MOESM1_ESM.pdf]
